# Sexual reproduction during diatom bloom

**DOI:** 10.1093/ismeco/ycae169

**Published:** 2025-01-07

**Authors:** Léa Prigent, Julien Quéré, Martin Plus, Mickael Le Gac

**Affiliations:** Ifremer, Dyneco, F-29280 Plouzané, France; Ifremer, Dyneco, F-29280 Plouzané, France; Ifremer, Dyneco, F-29280 Plouzané, France; Ifremer, Dyneco, F-29280 Plouzané, France

**Keywords:** temporal dynamics, harmful algal blooms, *Pseudo-nitzschia australis*, metatranscriptomic, sexual reproduction, life cycle

## Abstract

Phytoplankton supports food webs in all aquatic ecosystems. Ecological studies highlighted the links between environmental variables and species successions *in situ*. However, the role of life cycle characteristics on phytoplankton community dynamics remains poorly characterized. In diatoms, sexual reproduction creates new genetic combinations and prevents excessive cell size miniaturization. It has been extensively studied *in vitro* but seldom in the natural environment. Here, analyzing metatranscriptomic data in the light of the expression patterns previously characterized *in vitro*, we identified a synchronized and transient sexual reproduction event during a bloom of the toxic diatom species *Pseudo-nitzschia australis*. Despite the complexity of environmental conditions encountered *in situ,* sexual reproduction appeared to be the strongest differential gene expression signal that occurred during the bloom. The potential link between environmental conditions and the initiation of sexual reproduction remain to be determined, but sexual reproduction probably had a major impact on the bloom dynamic.

## Introduction

Phytoplankton plays a major ecological role. It has remarkable morphological and functional diversity and contributes to about half of Earth primary production [1]. Among this group, diatoms represent one of the most diverse and dominant protists [2, 3]. When environmental conditions favor high growth rate and low mortality, diatoms can temporarily proliferate to high densities (up to millions of cells per liter) and form a natural phenomenon called bloom [4]. Improving our understanding of algal blooms requires the ability to detect entire bloom events (from the start until the end), to identify the parameters that lead the causative species to transiently dominate the community and eventually be replaced by other species. Numerous studies have been carried out to identify the environmental factors that trigger the initiation, influence the magnitude and lead to bloom termination. Abiotic factors influencing species occurrence and dynamics include hydrodynamic processes, temperature, salinity and nutrient availability [5], whereas biotic factors include grazing, pathogenicity, parasitism, and competitive interactions within the microbial communities [5, 6]. Classically, temporal successions between phytoplankton functional groups is conceptualized using Margalef’s mandala that originally took into account turbulence and nutrients [7] and was later expanded by integrating twelve effect or response traits ranging from cell motility, pigment composition, to the ability to produce allelopathic compounds [8]. In dinoflagellates, life cycle characteristics, such as the ability to form resting cysts that may constitute “seed banks” have been considered as an important factor that may explain bloom dynamics across years [9]. In diatoms, life cycle is rarely considered as a factor influencing bloom dynamics.

Diatom life cycles consist in a diploid vegetative phase lasting months to years, during which cells divide mitotically, and in a sexual phase lasting a few days [10, 11]. The numerous mitotic divisions are associated with a cell size decrease imposed by the rigid silica frustule composed of two theca with the smaller fitting into the larger. During divisions, each of the two daughter cells retains one maternal theca and a smaller one is synthesized, leading to an average decrease in cell size throughout divisions. This cell size decrease could lead to death if cells reach a critical size incompatible with survival. Sexual reproduction generates bigger cells and is the only way to escape the miniaturization process. It may be initiated when cells reach the sexualization size threshold (SST) [12, 13] and is also density dependent, with sexualization occurring at high densities [14]. In *P. multistriata*, sexual reproduction has been initiated for cells between 40 and 55 μm, compared to a maximum cell size of ~80 μm [15]. Pennate diatoms are predominantly heterothallic and gamete formation occurs when two strains of opposite mating types (MT+ and MT−) come into contact under stable conditions [16]. Pheromones are involved in mating partner recognition in some species [17–19]. The initiation of sexual reproduction has been associated with a population wide growth arrest, even for cells not directly involved in sexual reproduction [14, 17, 20]. Following the initial contact, meiosis produces gametes that conjugate and produce a zygote. Zygotes expand into non-silicated cells, the auxospores, within which large initial vegetative cells are synthesized [13, 21]. Several studies have investigated diatom sexual reproduction *in vitro* using molecular approaches [22–27], *P. multistriata* having been used as a model to understand the molecular bases of pennate diatoms sexual reproduction [28]. Five mating related genes have been identified [26]. The MRP3 gene is expressed in MT+ strains and regulates the expression of MRP1 and MRP2 in MT+ strains and MRM1 and MRM2 in MT− strains. During mating type perception, at the initial stage of cell sexualization, the most upregulated genes in MT− were the endopeptidase cathepsin D, MRM2, the meiotic gene Rad51A1 and an uncharacterized gene 7488. In MT+, MRP1 was the most overexpressed gene in MT+ during sexualization [25]. Following the initiation of sexual reproduction, major transcriptional changes occurred in numerous metabolic functions such as photosynthesis, photoprotection, carbon assimilation and fatty acid metabolism [20].

Very few studies have reported observations of diatom sexual events in the natural environment. Sexual stages of *Pseudo-nitzschia* species in their natural environment have been observed and reported twice [29, 30]. In Europe, sexual stages of two species of *Pseudo-nitzschia* were detected in the Bay of Naples (Mediterranean Sea), where they accounted for 9.2 and 14.3% of the total number of cells of *P. cf. delicatissima* and *P. cf. calliantha,* respectively [29]. Another massive sex event involving *P. australis* and *P. pungens* had been reported along the Washington coast (Pacific Ocean). Auxospores were detected for about 3 weeks and accounted for up to 59% of the *P. australis* population at the end of the bloom [30]. Sexual reproduction stages have also been observed for *Fragilariopsis kerguelensis* and *Corethron criophilum* in the Southern Ocean [31, 32].

Here, we showed that synchronous and transient sexual reproduction is the strongest differential gene expression signal that occurred during a bloom of the toxic diatom *P. australis*, producer of domoic acid*,* and that it was probably a major determinant of the bloom dynamic. These results were obtained using a metatranscriptomic approach and built upon markers of sexual reproduction as well as global expression patterns identified during *P. multistriata in vitro* sexual reproduction [20].

## Materials and methods

### Field sampling and metatranscriptomic samples

From 4 April to 24 April 2017, duplicate samples were collected over 14 days during a *P. australis* bloom (Supplementary Table 1). Subsurface water samples were collected from shore at a single site (48.3601°N; −4.5531°E) in the Bay of Brest (Brittany, North-West of France; Supplementary Fig. 1) around high tide (+/− 2 h). Samples were filtered (6–15 liters, Supplementary Table 1) onto polycarbonate filters (10 μm, 47 mm) using a peristaltic pump before being frozen in liquid nitrogen in ribonucleic acid (RNA) later (Fisher Scientific, Illkirch, France) and stored at −80°C before extraction.

### Environmental data

High tide nitrate, phosphate and silicate surface concentrations (μmol/L, weekly data frequency) were obtained from the SOMLIT monitoring network (https://somlit.fr) at the Brest-Portzic station located 150 meters from our sampling station. High frequency (20 min) surface temperature (°C), turbidity (NTU) and chlorophyll fluorescence (Fluorescein Fluorescence Units, FFU) were obtained from the nearby COAST-HF-MAREL-Iroise buoy (48.3579°N, −4.5517°E) [33].

Average photosynthetically active radiations (PAR expressed in μmol photon/m^2^/s) reaching the sea surface at the sampling station and daily rainfall values (mm) were extracted from the Météo-France AROME numerical model (1.3 km resolution) [34]. Finally, hourly tide levels were provided by the National Hydrographic Service (SHOM, REFMAR dataset, http://dx.doi.org/10.17183/REFMAR, Brest tide gage).

### Ribonucleic acid extraction, library preparation, and sequencing

Total RNA was extracted by sonicating filters on ice (Vibra-cell 75 115, Bioblock Scientific, Illkirch, France) for 30 seconds at 35% intensity in LBP buffer (Macherey-Nagel, Duren, Germany). Extraction was performed using NucleoSpin® RNA Plus kit (Macherey-Nagel) following the manufacturer’s protocol. Library preparation was performed using the Illumina mRNA TruSeq stranded kit starting from 0.5 μg of total RNA. Library preparation failed for two samples. A total of 26 samples were paired-end sequenced using 2 × 150 bp cycles on Illumina Novaseq6000 at the GeT-PlaGe France Genomics sequencing platform (Toulouse, France). To avoid batch effects, samples were randomized for RNA extraction, library preparation and sequencing. Generated fastQ files have been deposited to ENA (Supplementary Table 1).

### Bioinformatic analysis

Prior to read mapping, raw reads quality were assessed using FastQC (http://www.bioinformatics.bbsrc.ac.uk/projects/fastqc/), and Trimmomatic (V. 0.33) [35], was used to trim ambiguous, low quality reads and sequencing adapters with parameters ILLUMINACLIP: Adapt.fasta: 2: 30: 10: 8 LEADING: 3 TRAILING: 3 MAXINFO:135:0.8 MINLEN: 80.

#### Community composition

Community composition was estimated using blastn to identify similarity between the forward trimmed reads and two reference databases. The first database, PR2 [18S ribosomal RNA (rRNA)] [36] was used to determine eukaryote relative abundance at the class level. The second one, Diat_barcode (*rbcL*) [37] was used to determine diatom relative abundance at the genus or species level. For each database, reads were considered if the hits displaying the minimum e-value all belonged to the same class (PR2, e-value ≤10^−70^), genus or species (Diat_barcode, e-value ≤10^−30^). The relative abundances were obtained by dividing the number of reads associated with each species, genera or class, by the total number of taxonomically assigned reads in each sample.

#### Gene expressions and functions analysis

##### Reference transcriptomes and alignment

Using the BWA-MEM aligner [38], trimmed reads were aligned to a metareference corresponding to the combination of 315 species specific reference transcriptomes, representing 213 unique genera [39]. It mostly corresponded to the resources developed during the Marine Microbial Eukaryotic Transcriptome Sequencing Project [40] with the addition of reference transcriptomes obtained for three *Pseudo-nitzschia* species (*P. australis*, *P. fraudulenta*, and *P. pungens*) based on local strains [41]. Samtools was used to discard reads displaying low quality alignments (MapQ<10), to remove read pairs that did not align to the same transcript and to generate the raw read count expression matrix [42]. Raw read counts corresponding to *P. australis* contigs were then extracted.

##### Gene expression dynamics

After preliminary analyses, the 24 April samples were excluded due to low relative abundances of *P. australis. T*ranscripts covered by <5 reads on average per day were discarded from the analyses. Overall gene expression variability among samples was explored following rlog transformation using a principal component analysis (PCA) as implemented in Deseq2 [43]. After checking their homogeneity, replicates were pooled by summing the expression matrices.

Pairwise differential expression (DE) was tested between sample groups (see results) using Wald tests (DESeq2) considering a negative binomial generalized linear model with a false discovery rate (FDR) set at qvalue = 0.05. DE transcripts were clustered based on expression profiles across samples using negative binomial models as implemented in MBCluster.Seq [44]. Different cluster sizes were visually inspected and clusters with similar profiles were merged. Gene expression is reported as the log2 fold change (log2FC) of the expression of a given transcript over the median expression considering all samples. It is calculated as: log2(2^Xi^/2^X-^) where Xi is the rlog transformation of the number of reads mapping to a given transcript for each sample i and X- is the rlog transformation of the median number of reads mapping to a given transcript for all samples.

The relationship between gene expression (rlog) and environmental factors (PAR, salinity, temperature, fluorescence, turbidity, and tidal amplitude) was analysed using a canonical correlation analysis (CCA) as implemented in the vegan R package [45]. Rainfall and nutrients were excluded from this analysis due to very low rainfall and low frequency nutrient analyses, respectively.

##### Functional annotation

The reference transcriptome of *P. australis* was previously annotated [41]. It was based on sequence similarity with the manually curated Uniprot database as inferred using blastx and considering e-value <10^−3^. Contigs were classified in Gene Ontology (GO) categories (http://geneontology.org/). Overrepresentation of GO categories in the clusters was tested using Fisher Exact tests followed by FDR correction with a q-value threshold set at 0.05. Fisher Exact tests odds ratios (ORs) were reported. Only GO categories containing more than five DE transcripts in a given cluster were considered. To take account GO redundancy (i.e. a similar set of genes can be found in different GO categories), GO categories displaying an overlap coefficient $\frac{GOi\cap GOj}{\mathit{\min}\left( GOi, GOj\right)}$ > 0.8 (where GO_j_ is the size of the GO category j) were clustered [41].

Genes belonging to cluster 6 (see results) were further annotated based on homology with proteins of identified function in the NCBI nr database using blastx (e-value <10^−3^). Genes involved in domoic acid production and related to mating type locus were annotated based on homology (tblastn) with *P. multiseries dabA*, *dabB*, *dabC*, and *dabD* genes [46] and *P. multistriata* MRM1, MRM2, MRM3, MRP1, and MRP2 genes [26, 41], respectively. Homolog of the silicon transporter sit1 was also manually annotated [47]. Annotations are reported in Supplementary Table 2.

Genes differentially expressed during *P. multistriata* crossing experiments were obtained from [20] (datafile_S1). These expression levels were obtained 1 hour after parental cells of opposite MT had been mixed (T1), 24 hours after initial mixing (T2) and five days after initial mixing (T3). At T2, ~4% of parental cells had formed gametes. At T3, gametes, zygotes, auxospores, and F1 cells represented 7% of the total population. Homology with *P. australis* genes was identified using tblastx (e-value <10^−10^). The association between genes belonging to the *P. australis* gene clusters displaying differential gene expression *in situ* (see Results) and *P. multistriata* genes displaying DE at T1, T2, and T3 during crossing experiments was tested using Fisher Exact tests.

## Results

### Community composition

**Figure 1:**
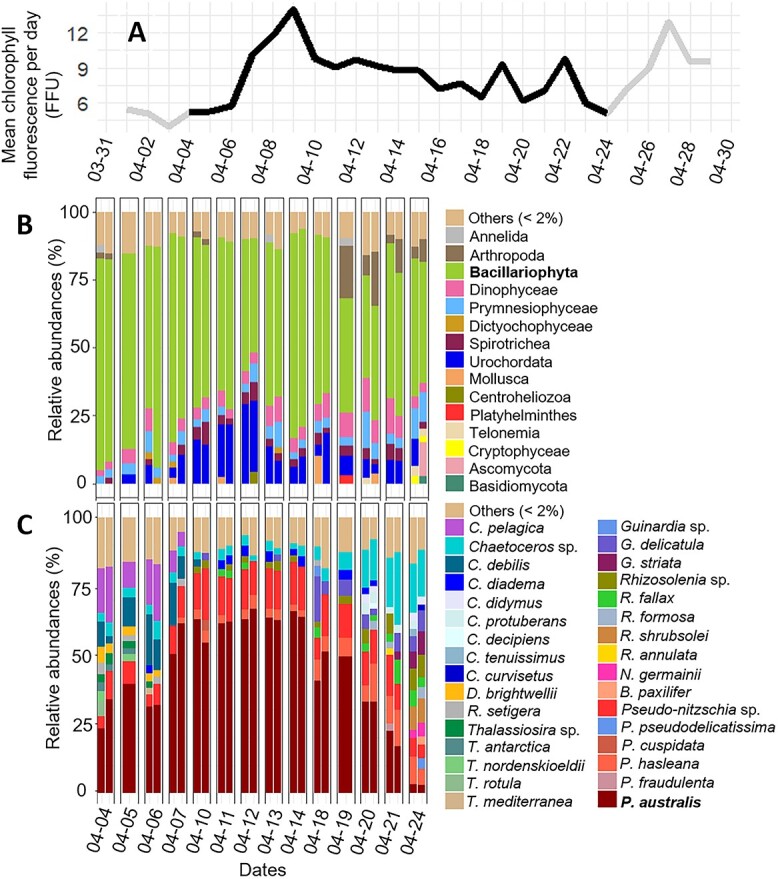
A. Temporal evolution of the mean chlorophyll fluorescence per day between 2017-04-01 and 2017-04-29. The survey period is shown in black. B. Relative abundances of the class community composition during the monitoring. C. Relative abundances of the diatom community composition during the monitoring (C: genus *Cerataulina* then *Chaetoceros*; D: *Ditylum*; T: *Thalassiosira*; G: *Guinardia*, R: *Rhizosolenia*; N: *Navicula*; B: *Bacillaria*; P: *Pseudo-nitzschia*). The category others regroup class, genus, or species <2%.

At the beginning of April 2017, a strong increase in chlorophyll fluorescence indicated the first massive phytoplankton development at the sampled station (Fig. 1A; Supplementary Fig. 2). This bloom lasted until the end of the month. The eukaryote community relative abundances at the Class level indicated that diatoms (*Bacillariophyta*) strongly dominated the community throughout the bloom, with relative abundances ranging from 38% to 81% (Fig. 1B). *Dinophyceae*, *Prymnesiophyceae*, and *Spirotrichea* were present but <14%. *Arthropoda*, mainly copepods, appeared at the end of the bloom, reaching a maximum relative abundance of 20% on 19 April (Fig. 1B; Supplementary Fig. 3). The species dominating the bloom was *P. australis*. It represented ~20% of the diatoms at the beginning of the survey, increased to more than 60% between 7 and 14 April and decreased afterward (Fig. 1C). At the beginning of the bloom, the accompanying species mostly belonged to the genus *Cerataulina*, *Chaetoceros*, and *Thalassiosira*, while at the end, relative abundances of *Chaetoceros*, *Guinardia*, and *Rhizosolenia*, but also of *P. hasleana* increased.

### Environmental conditions

**Figure 2:**
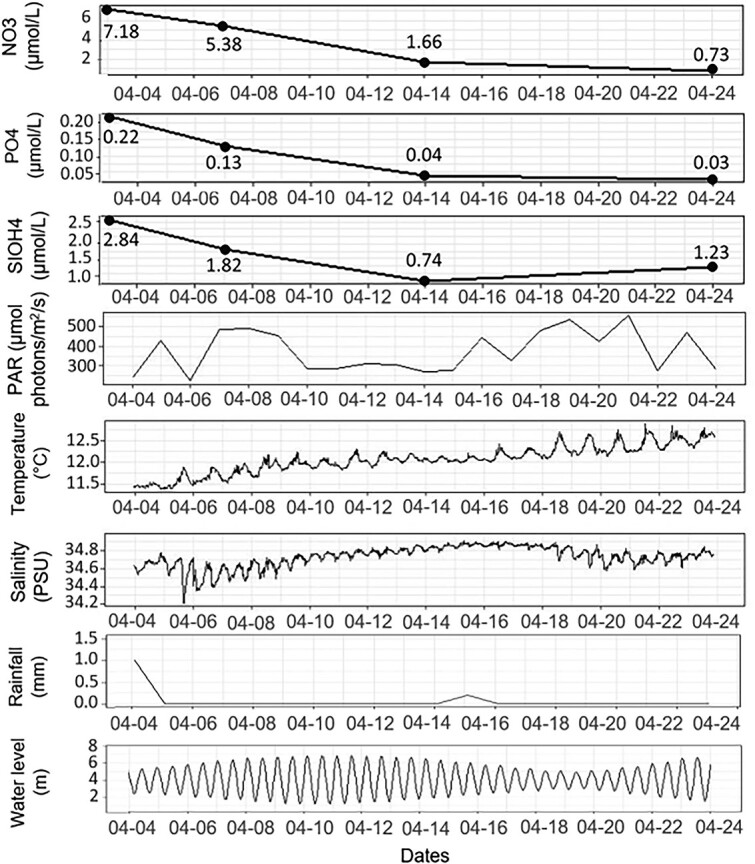
Temporal evolution of abiotic parameters during the sampling period for nutrients: Nitrate (NO3), phosphate (PO4), and silicate (SiOH4), average photosynthetically active radiation, temperature, salinity, daily rainfall, and water level.

During the sampled period, the environment reflected typical spring transitions with a regular decrease in nutrient concentrations (from 7.2 μmol/L to 0.7 μmol/L for nitrates; from 0.2 μmol/L to 0.03 μmol/L for phosphates and from 2.84 μmol/L to 1.23 μmol/L for silicates) associated with a slight increase in salinity (Fig. 2). Such a trend resulted from a decrease in riverine flow due to low rain throughout the period. The weather was mostly sunny, and resulted in a regular increase in sea water temperature, from an average of 11.5°C on 4 April to 12.5°C on 24 April. In parallel, the average PAR values showed variability, decreasing for some days over the second week of monitoring with an average of 293 μmol photons/m^2^/s. Sea water temperature was also affected by the night-day cycle, with an increase in temperature during the day. Salinity was affected by the semi-diurnal tidal variation between low and high tide, with low salinity at low tide. Finally, temperature and salinity were affected by changes in tidal amplitude, with higher diurnal temperature and semi-diurnal salinity fluctuations when tidal amplitudes were minimal (4 to 6 April and 18 to 22; Fig. 2).

### Gene expression dynamics change over the bloom period

**Figure 3:**
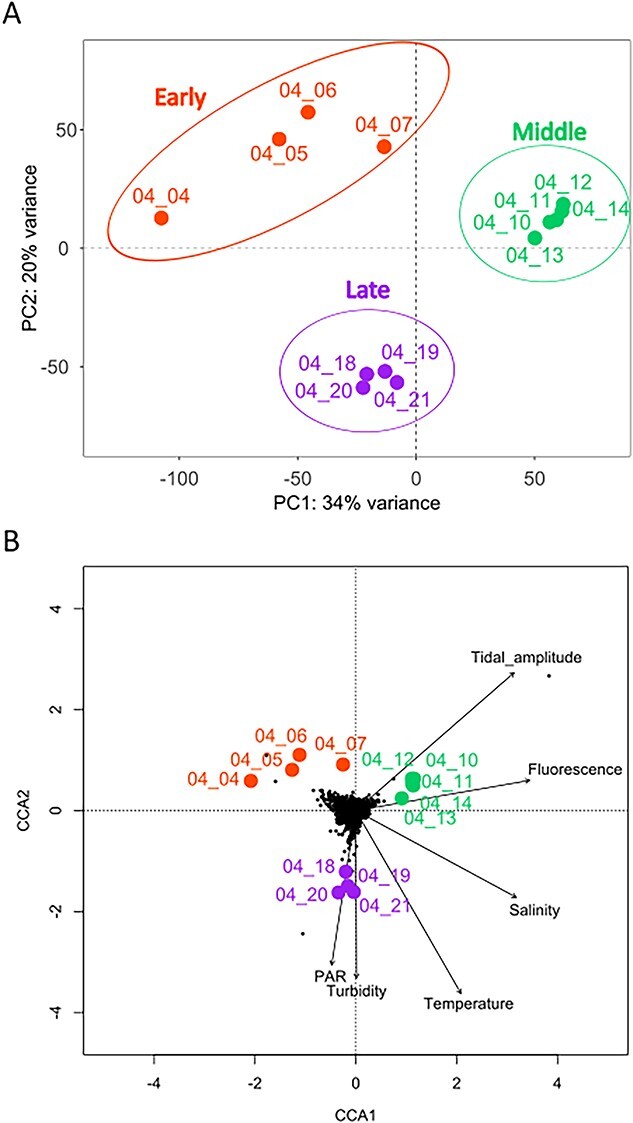
Gene expression variability and relation to environmental conditions. A. PCA of normalized *P. australis* gene expression (25 140 genes) profiles for each sample. The three sample groups are identified by circles. B. Canonical correspondence analysis of *P. australis* gene expression in relation to environmental conditions. Samples are indicated by colored dots and text. Black dots correspond to individual genes and black arrow to environmental parameters.

To determine gene expression dynamics of *P. australis*, meta-transcriptomic reads aligning specifically on the *P. australis* reference transcriptome were extracted. To visualize gene expression variability between samples, a PCA was performed, using the 25 140 *P. australis* expressed genes (Supplementary Table 3). The two first PCA components explained 54% of the gene expression variability (Fig. 3A). Three groups of samples displaying similar gene expression were identified. They correspond to samples from 4 to 7 April, 10 to 14 April, and 18 to 21 April. These three groups of samples, hereafter named early, middle and late groups, respectively, were obtained in slightly different environmental conditions (Fig. 3B). The early group was associated with slightly lower temperature, salinity and chlorophyll fluorescence. The middle group of samples was associated with higher tidal amplitudes and chlorophyll fluorescence. The late group was associated with higher temperature, PAR, and turbidity.

**Figure 4:**
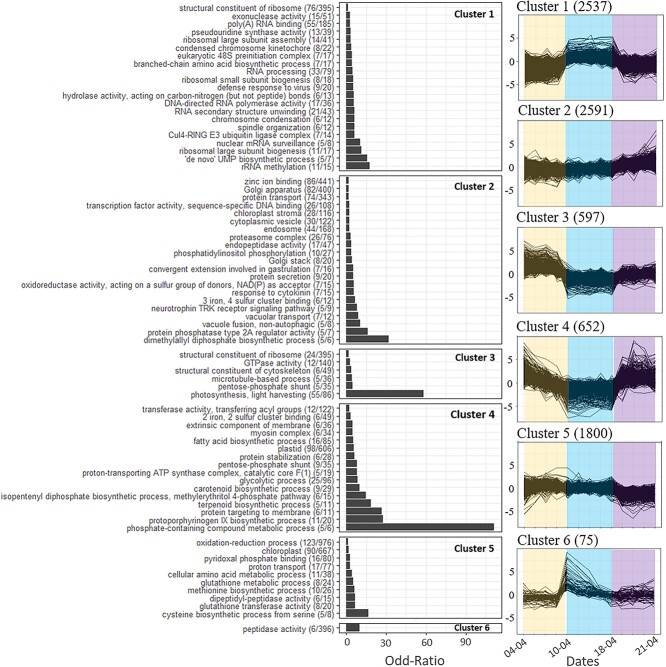
Clusters of *P. australis* genes displaying dynamic expression during the bloom. Right side: clusters of DE genes displaying similar gene expression dynamics (expressed as log2FC) across samples. The number of transcripts belonging to each cluster is indicated in parenthesis, colors indicate early, middle and late sample groups. Left side: Overrepresented biological processes in each cluster (GO terms). GO terms (y axis) were presented based on their odd-ratio (x axis). For each GO term, the number of transcripts belonging to the cluster over the total number of transcripts present in *P. australis* transcriptome is indicated in parenthesis.

Differential gene expression was tested between these three groups. A total of 8252 genes were identified as differentially expressed between the three groups of samples. These genes were grouped into 6 clusters based on gene expression changes across samples (Fig. 4). The clusters contained between 75 (cluster 6) and 2591 (cluster 2) genes. Overall, these gene clusters displayed pronounced gene expression differences between the early, middle and late groups of samples, with major shifts in gene expression occurring on 10 and 18 April.

The over-representation of gene functions was analyzed in the 6 clusters. At the beginning of the bloom (from 4 to 10 April), numerous genes involved in photosynthesis were over-expressed (Fig. 4, cluster 3, Supplementary Table 4). In addition, although decreasing during this first phase, there was also an over-expression of genes involved in energy production and storage (Fig. 4, cluster 4, Supplementary Table 4), as well as in pigment and fatty acid biosynthesis (cluster 4, Supplementary Table 4). During the second phase (from 10 to 14 April), genes involved in ribosomal biogenesis and protein synthesis as well as in chromosomal organization were over-expressed (Fig. 4, cluster 1, Supplementary Table 4). During the late phase (from 14 to 21 April), expression slightly increased for genes related to photosynthesis (Fig. 4, cluster 3, Supplementary Table 4) and a more pronounced increase was detected for genes related to energy production and storage, as well as involved in the biosynthesis of pigments and fatty acids (Fig. 4, cluster 4, Supplementary Table 4). There was a very strong overrepresentation of genes involved in “phosphate-containing compound metabolic process”, which may indicate tight regulation of phosphate utilization during the late phase. It was also characterized by an under-representation of various functions related to the cellular redox balance (Fig. 4, cluster 5, Supplementary Table 4), suggesting a perturbation of the cellular redox balance toward the end of the bloom.

Throughout the entire bloom, there was a moderate and rather constant gene expression increase for several processes related to excretion and more specifically to protein excretion (Fig. 4, cluster 2; Supplementary Table 4). Associated with this, there was a strong overrepresentation of genes involved in the biosynthesis of dimethylallyl diphosphate, an important precursor of isoprenoids synthesis, including carotenoids. It is also important to note that the dimethylallyl diphosphate is a domoic acid precursor.

### Sexualization of *Pseudo-nitzschia australis* cells

Between the early and middle phases, genes belonging to cluster 6 displayed a transient high over-expression. In this cluster, the only over-represented GO category is “peptidase activity” which may indicate a metabolic shift. In order to understand the major changes that occurred on 10 April, the annotation of individual genes belonging to cluster 6 were analyzed.

**Figure 5:**
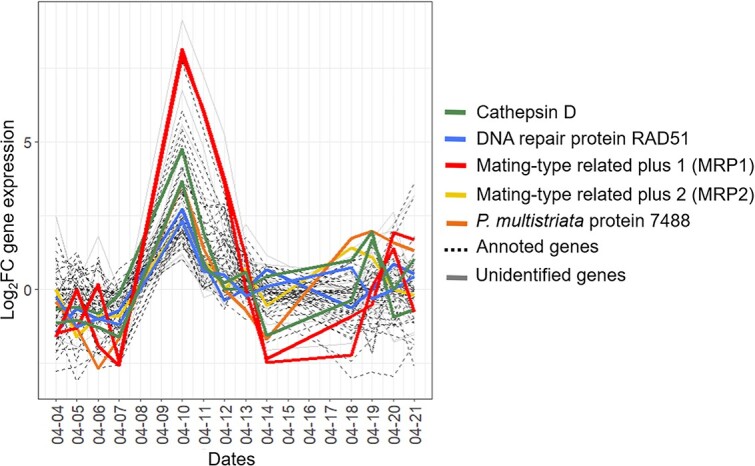
Temporal trends of expression for genes belonging to cluster 6. Genes related to the initiation of sexual reproduction are in colors, genes with other annotated functions are indicated with a dotted black line, and unannotated genes are in gray.

Out of the 75 genes belonging to cluster 6, 28 could not be annotated (Supplementary Table 2). The two annotated genes displaying the highest over-expression levels on 10 April were homologous to the “mating type related plus 1” gene (MRP1, log2FC of 8.1 and 7.9; Fig. 5) identified in *P. multistriata*. This gene had been shown to be the most over-expressed gene in MT+ cells during sexualization [25]. Moreover, homologs of three out of the four most DE genes during MT− sexualization were also in the cluster 6. It was the case of two Rad51 homologs (log2FC of 2.7 and 2.3; Fig. 5), two cathepsin D (log2FC of 4.8 and 3.7; Fig. 5) homologs and one homolog of *P. multistriata* protein 7488 (log2FC of 3.6; Fig. 5). The homolog of the fourth gene displaying high DE during MT− sexualization, MRM2, did not belong to cluster 6. However, it also displayed a transient increase in expression on 10 April (log2FC = 2). Another mating type related gene, MRP2, also belonged to cluster 6 (Log2FC = 2.1 on 10 April). Of the two remaining mating type related genes MRP3 displayed a rather stable expression level throughout the bloom and MRM1 was not expressed.

### Expression of genes related to sexual reproduction during the bloom

**Figure 6:**
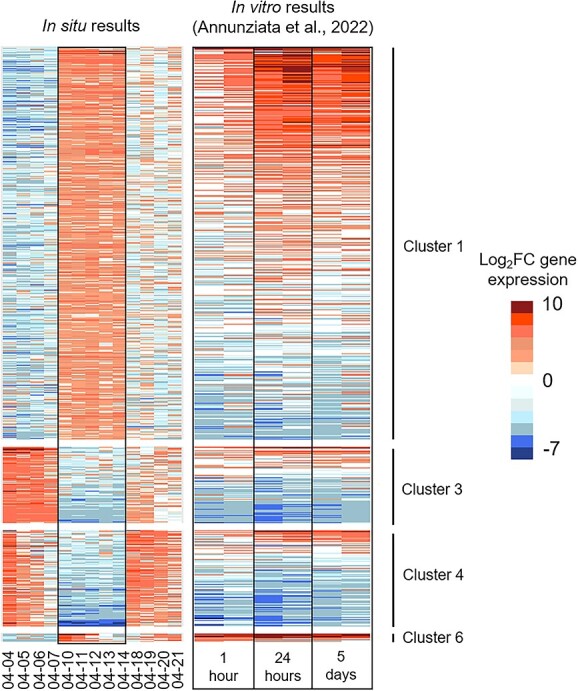
Comparison of the expression levels of *P. multistriata* genes identified as differentially expressed at one hour, 24 hours, and 5 days post-crossing compared to mating type + and - cells *in vitro* (from [20]), with their homologs identified in the *P. australis* reference transcriptome and belonging to clusters 1, 3, 4 and 6 *in situ*. For each *in vitro* time point, the two columns indicate the expression level of the cross against MT+ and MT− strains, respectively. Gene names and log2FC indicated in Supplementary Table 5.

The pattern of expression presented above suggested the initiation of a sexual reproduction event on 10 April. In order to test this hypothesis, we compared the expression of *P. australis in situ*, to the gene expression of the closely related species *P. multistriata* characterized *in vitro* during sexual reproduction. During *in vitro* experiments in *P. multistriata*, from initial contact to F1 generation, sexual reproduction lasted several days. For genes belonging to cluster 6 the odds of having homologs up-regulated one hour after initial parental cells contact during *P. multistriata in vitro* experiment were 24 times higher than randomly expected (two-sided Fisher exact test, Odd-Ratio = 24.4, P-value <2.2e^−16^; 24 up-regulated homologs out of 38 belonging to cluster 6; Fig. 6; Supplementary Table 5). This clearly added an argument in favor of *P. australis* sexual reproduction initiation *in situ* around 10 April. To determine whether the abrupt changes in gene expression observed following 10 April could be the result of sexual reproduction, genes belonging to cluster 3 and 4 (i.e. genes down-regulated during the second phase of the bloom) and to cluster 1 (i.e. genes up-regulated during the second phase of the bloom) were compared to *P. multistriata* genes displaying down and up regulation during *in vitro* sexual reproduction, respectively. For genes belonging to cluster 3 or 4, odds of having homologs down-regulated 1 hour, 5 hours and 5 days after initial parental cells contact during *P. multistriata in vitro* experiment were 7, 8 and 7 times higher than randomly expected, respectively (two-sided Fisher exact test, Odd-Ratio = 6.96, 8.23, 7.37, P-values <2.2e-16; 280/791, 400/791 and 304/791; Fig. 6; Supplementary Table 5). Moreover, the genes most strongly down-regulated in clusters 3 and 4 also appear to be strongly down-regulated during *in vitro* experiments (Fig. 6; Supplementary Table 5). Nevertheless, there were 208 genes belonging to clusters 3 and 4 that tended to be up-regulated during *in vitro* experiments (average log2FC > 0). Functions related to ribosome, proteolysis, one-carbon metabolic process and phagocytosis were overrepresented in these 208 genes compared to all the genes belonging to clusters 3 and 4 (Supplementary Table 6).

To a lesser extent, genes up-regulated during the middle bloom period also tend to be up-regulated from a few hours to a few days after the initiation of *P. multistriata* sexual reproduction *in vitro*. For genes belonging to cluster 1, odds of having homologs up-regulated 1 hour, 5 hours, and 5 days after initial parental cells contact during *P. multistriata in vitro* experiment were 1.3, 2.5 and 2.5 times higher than randomly expected, respectively (two-sided Fisher exact test, Odd-Ratio = 1.34, 2.48, 2.49; P-value = .002, < 2.2e-16, < 2.2e-16; 153/1804, 520/1804 and 401/1804; Fig. 6; Supplementary Table 5). Despite this strong correspondence, 604 genes tend to be up-regulated during the middle phase *in situ* (cluster 1) but down-regulated during *in vitro* experiments (average log2FC < 0). Highlighting differences between sexual reproduction *in vitro* and *in situ*, functions related to cell division, mitosis and chromosome condensations (Supplementary Table 7) were strongly overrepresented in these 604 genes compared to all the genes belonging to cluster 1.

### Expression of toxins biosynthesis genes

**Figure 7:**
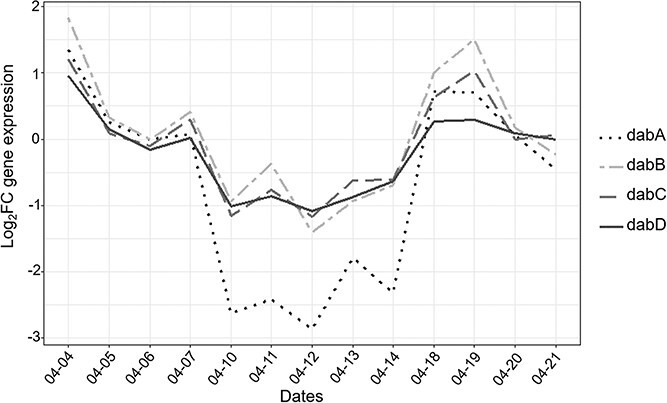
DA biosynthesis gene expression dynamics in *P. australis*. The expression of the *P. australis* genes *dabA*, *dabB*, *dabC*, and *dabD* during the time course of the bloom is indicated.

The four genes (*dab*ABCD) identified as involved in the biosynthesis of DA in *P. multiseries* [46] displayed in our dataset a drop in expression during the early phase, a stable low expression during the middle phase followed by a further increase during the late phase (Fig. 7). Among these genes, two (*dabA* and *dabB*) showed a significant difference in expression during the survey and belonged to cluster 4.

## Discussion

Results presented above show strong evidence of synchronous and transient sexual reproduction during a bloom of the toxic diatom *P. australis*.

First, a transient over-expression of a few tens of genes highlighted the sexualization of the *P. australis* vegetative cells. Indeed, homologs of the five most upregulated genes during *in vitro* sexualization of *P. multistriata* displayed high expression levels at a single sampling date during our survey. This was true for the mating type plus related gene MRP1, the most overexpressed gene in MT+ during the sexualization as well as for the mating type minus related gene MRM2, the endopeptidase Cathepsin D, the meiotic gene Rad51 and the uncharacterized gene 7488, which are the most overexpressed genes in MT− during the *P. multistriata* sexualization [25]. More generally, besides these marker genes, the transiently up-regulated genes in *P. australis* corresponded to the genes upregulated one hour after opposite mating type contact during *P. multistriata* experiments [20]. This transient signal, peaking on a single day and quickly decreasing over the next couple of days, was a clear indication that the initiation of sexual reproduction was synchronized at the population scale. It does not mean that all cells entered into sexual reproduction. Even during sexual reproduction experiments, only a portion of the parental cells, ranging from 10% to 30%, reproduced sexually simultaneously [14, 20]. However, the extremely strong up-regulation (from 0 reads up to several thousands of reads) of the genes mentioned above, indicated that the proportion of cells simultaneously entering sexual reproduction was sufficient to induce a population wide signal. *In situ* reports of sexual reproduction in *Pseudo-nitzschia* species are scarce, but are in line with *in vitro* observations. Sexual stages detected in the Bay of Naples (Mediterranean Sea) accounted for 9 and 14% of the total number of cells of *P. cf. delicatissima* and *P. cf. calliantha*, respectively [29]. Another massive sex event, involving *P. australis* and *P. pungens* had been reported along the pacific coast of North America with auxospores accounting up to 59% of the *P. australis* population at the end of the bloom [28]. These varying time scales mean that studying this process in its natural environment is very difficult [48]. During experiments, sexual reproduction was induced by mixing MT+ and MT− strains under non-limiting conditions [16, 20, 21, 25, 26], with higher rates of sexual reproduction occurring when cells were in exponential phase [14]. *In situ*, cells of opposite mating types were co-occurring before sexual reproduction initiation, so one may wonder what the signal triggering synchronous sexualization was. *In vitro* studies have demonstrated that sexual initiation depends upon the cell sizes. In diatoms, cell size tends to decrease through the successive mitotic divisions spanning several months to several years until it reaches the SST, a cell size below which sexual reproduction may be induced [12, 13]. Based on cells size patterns along a multi annual *P. multistriata* time series, it has been proposed that sexual reproduction in the field could occur every three years [49]. Given a sexual event lasting for a few days, this may explain why reports of sexual reproduction in the field remain extremely rare. Sexualization also depends upon cell densities and high cell density is required to favor cells physical proximity and sex initiation [14, 15]. This is in agreement with the results of the present study, where sexualization occurred when *P. australis* cell densities were maximum. During the study period, changes in abiotic environmental conditions were recorded, but not specific change could be identified as triggering sexualization.

Second, following this transient expression pattern, a massive shift in *P. australis* gene expression was recorded *in situ*. This shift lasted for a few days and the observed expression pattern corresponded to the one observed from a few hours to a few days after opposite mating type initial contact during *P. multistriata* experiments, when gametes, zygotes and auxospores were observed [20]. Gene expression similarity was especially strong for downregulated genes, with low expression of genes involved in photosynthesis, glycolysis, phosphate metabolism, fatty acid, and carotenoid biosynthesis. This could indicate that down regulation of these genes may be the hallmark of sexual reproduction in *Pseudo-nitzschia*, independently of environmental conditions. For instance, prior to sexual reproduction, *P. multistriata* cells tend to accumulate lipid droplets to store energy and potentially transfer it to F1 cells [14, 20, 22]. Moreover, down-regulation of photosynthesis related genes was described as a method to decrease the input of photochemical energy in a way to protect cell health during the sexual phase [20]. However, during the *P. australis* bloom, PAR was higher during the late bloom phase but also to a lesser extend during the early phase, coinciding with higher expression of photosynthesis related genes. In the same vein, light intensity was high enough during these two phases to be considered as potentially stressful and could have triggered the biosynthesis of photoprotection pigments such as carotenoids [50]. As a result, it is difficult to determine whether DE of photosynthesis and carotenoid biosynthesis genes was caused by changes in light conditions, sexual reproduction or both. Of specific interest was the down-regulation of *dab* genes, suggesting lower domoic acid production during sexual reproduction [46]. A significant overlap was also detected between genes up-regulated *in vitro* and *in situ.* However the overlap was less strong than for down-regulated genes, which might suggest that, overall, up-regulated genes are less specific to sexual reproduction, and perhaps more dependent upon environmental conditions, than down-regulated ones. Several *in vitro* studies showed that a population wide growth arrest, lasting a few days, followed the initiation of sexual reproduction in *P. multistriata*, even for cells not directly involved in sexual reproduction [14, 20, 21]. This growth arrest has also been identified *in vitro* in other pennate diatoms [51]. *In situ*, observed cell densities are the result of a balance between cell division, mortality and hydrodynamic dilution. It is therefore difficult to determine whether a growth arrest took place during the *P. australis* bloom. However, during the sexual reproduction event, both chlorophyll fluorescence (a proxy of primary producers biomass) and *P. australis* relative abundances remained high. In the study zone, tide is by far the strongest hydrodynamic force [52]. As a result, tidal amplitude may be considered as a good proxy of bloom dilution. During the sexual reproduction event, tidal amplitude, and as a result hydrodynamic dilution, were high. The maintenance of high levels of chlorophyll fluorescence and *P. australis* relative abundances was in favor of a maintenance of *P. australis* active growth. Of special interest was the fact that, among the genes displaying high expression during the sexual reproduction event *in situ*, but not *in vitro*, there was a strong over-representation of genes associated with cell division, mitosis and chromosome condensations. This is a further indication that, contrary to what has been observed *in vitro*, active growth may be maintained during sexual reproduction *in situ* [53, 54].

After the end of the sexual reproduction event, *P. australis* abundance quickly dropped before virtually disappearing in a matter of a few days. This occurred despite a strong decrease in tidal amplitude, and thus of hydrodynamic dilution, and could indicate a *P. australis* growth arrest that resulted in bloom termination. *In vitro*, *P. multistriata* growth rate tends to increase again after the sexual reproduction growth arrest [20], nonetheless in the few events characterized during environmental blooms, *Pseudo-nitzschia* cell densities quickly dropped following sexual reproduction events [29, 30]. In the present case, a transient increase in copepod relative abundance occurred the day after *P. australis* abundances started to drop. This may have accelerated bloom termination through grazing, but, because of this time lag, was unlikely to be the main origin of bloom termination. Beside slight changes in environmental conditions, no specific factor could be identified as explaining the bloom demise. However, during this last bloom phase, decreased expression of genes involved in the cellular redox balance and more specifically related to glutathione was identified. Glutathione being one of the most abundant antioxidants in cells, this expression pattern may be indicative of an oxidative stress, resulting from an imbalance between reactive oxygen species generation and antioxidant capacity [55]. Such a stress could lead to cell senescence [56, 57] that could explain the termination of the bloom. In the absence of any straightforward biotic or abiotic environmental change, it is difficult to determine whether the triggering mechanism could be environmental, or the end of the sexual reproduction event, with cell senescence of the remaining small cells that did not reproduce sexually.

## Conclusion

This study identified a transient and synchronous sexual reproduction event in the toxic diatom *P. australis in situ*. Gene expression patterns associated with sexual reproduction of a closely related species had been extensively studied *in vitro*. Thanks to these studies, it was possible to use gene expression patterns as markers of sexual reproduction. Based on the expression of a handful of marker genes as well as on global expression patterns, it appeared that vegetative cells sexualization occurred on a given day and that sexual reproduction lasted for less than a week. This corresponded the timing observed *in vitro*. The relationship between environmental condition and the initiation of sexual reproduction remained to be determined but it was probably initiated following high cell densities with cell sizes below the SST. Despite the complexity of biotic and abiotic environmental conditions encountered *in situ* and the genetic heterogeneity of *P. australis* populations, sexual reproduction appeared to be the strongest differential gene expression signal that occurred during the bloom. As a result, it was probably a major determinant of bloom dynamics, with potential impact on growth rate and bloom’s demise. The dynamic of a given species in microbial communities is mostly thought to be determined by the abiotic conditions acting on cell divisions as well as by biotic interactions that may modulate resources accesses and or mortality rates. The present study highlighted that life cycle events, such as sexual reproduction, may be a key parameter determining microbial community dynamics *in situ*.

## Supplementary Material

Sfig1_New_ycae169

Supplementary_figure2_ycae169

Supplementary_figure3_ycae169

Supplementary_Table_1_ycae169

Supplementary_table_2_ycae169

Supplementary_table_3_ycae169

Supplementary_table_4_ycae169

Supplementary_Table_5_ycae169

Supplementary_table_6_ycae169

Supplementary_table_7_ycae169

List_of_supplementary_tables_and_figures_ycae169

## Data Availability

Raw reads have been deposited to SRA with accession numbers indicated in Supplementary Table 1. Read counts and gene annotations are available in Supplementary Tables 2 and 3.
