# Supplementary figures and images for: Sexual reproduction during diatom bloom
Source: ISME Commun. 2025 Jan 7;5(1):ycae169. doi: 10.1093/ismeco/ycae169 (PMC11749564; doi:10.1093/ismeco/ycae169)

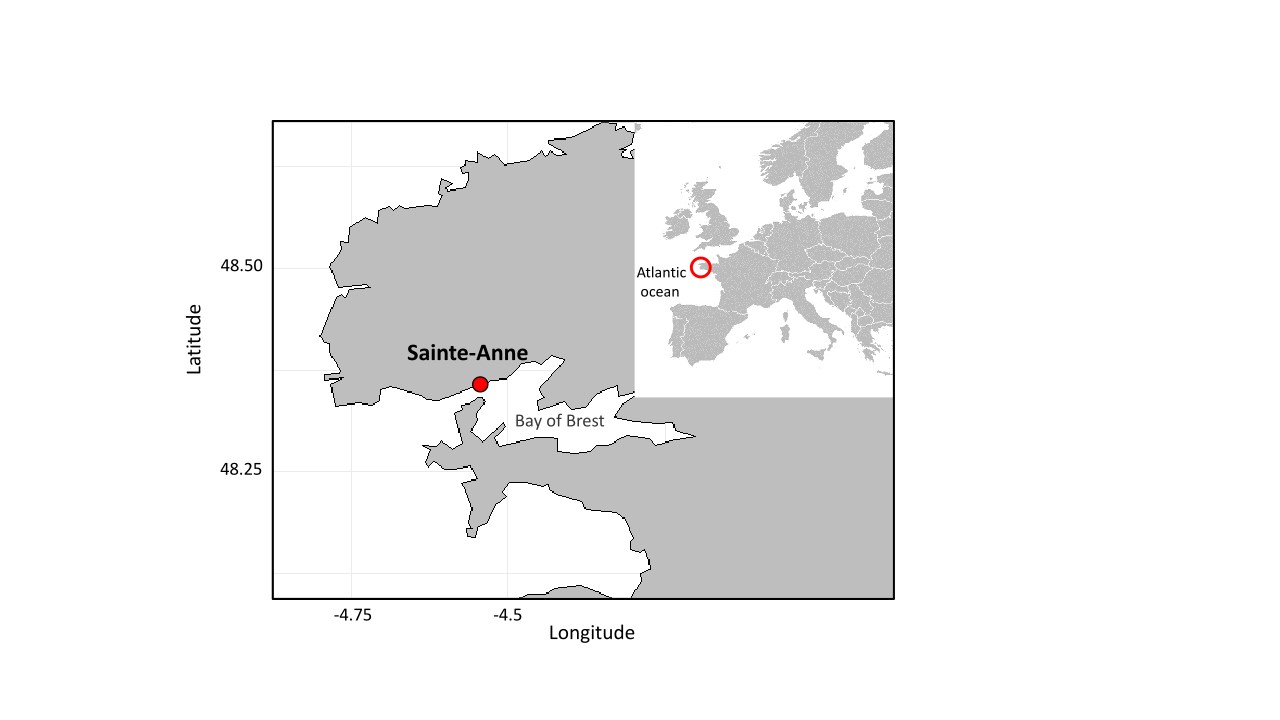

Supplement: Sfig1_New_ycae169 [file sfig1_new_ycae169.jpeg]

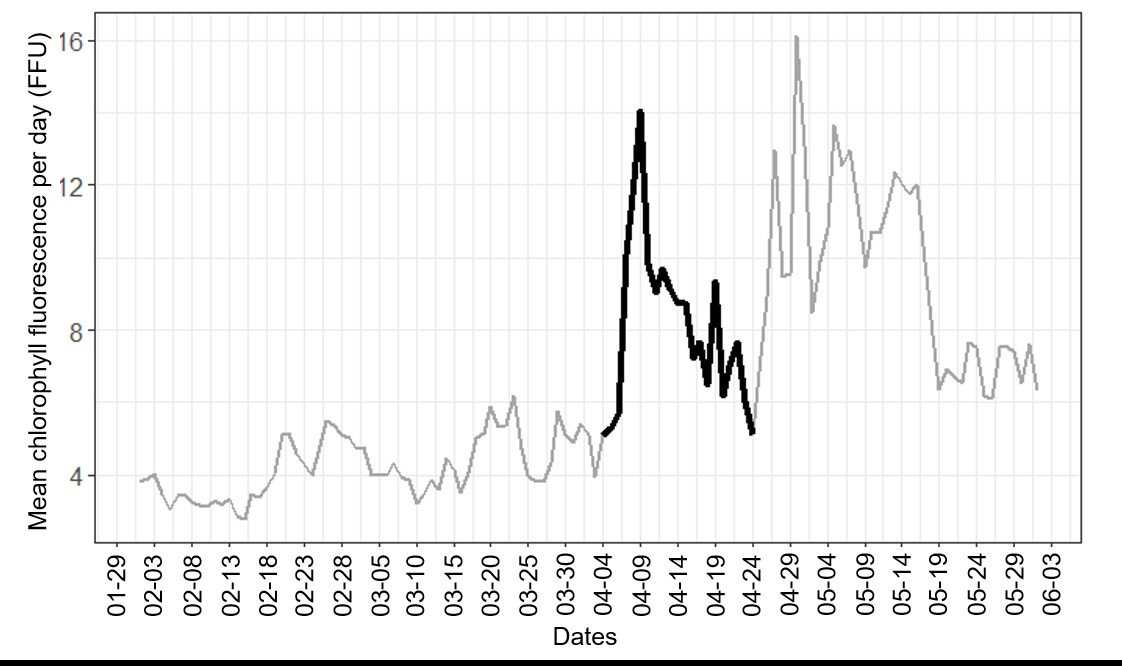

Supplement: Supplementary_figure2_ycae169 [file supplementary_figure2_ycae169.jpeg]

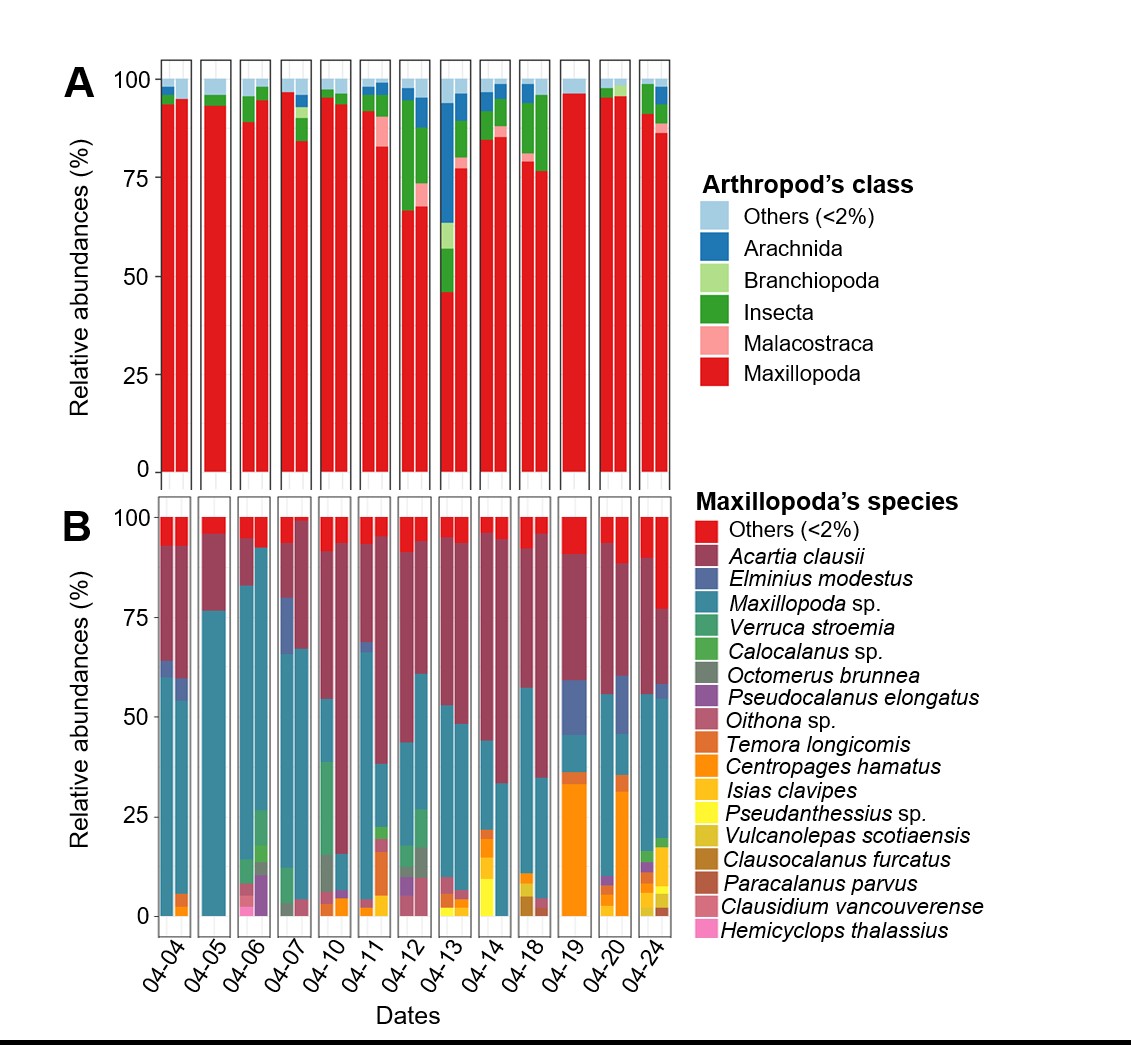

Supplement: Supplementary_figure3_ycae169 [file supplementary_figure3_ycae169.jpeg]
