# Supplementary material for: Sexual reproduction during diatom bloom
Source: ISME Commun. 2025 Jan 7;5(1):ycae169. doi: 10.1093/ismeco/ycae169 (PMC11749564; doi:10.1093/ismeco/ycae169)
Supplement: Supplementary_table_4_ycae169 [file supplementary_table_4_ycae169.pdf]

Broad function	Related GO categories
Photosynthesis	"Photosynthesis, light-harvesting"
Energy production and storage	“Glycolytic process”, “Pentose phosphate shunt”, “Fatty acid biosynthesis”, “Proton-transporting ATP synthase complex”
Pigment and fatty acid biosynthesis	“Terpenoid biosynthetic process”, “Fatty acid biosynthetic process”, “Carotenoid biosynthetic process”, “Protoporphyrinogen IX biosynthetic process”, “Isopentenyl diphosphate biosynthetic process, methylerythritol 4-phosphate pathway”
Ribosomal biogenesis and protein synthesis	“DNA-directed RNA polymerase activity”, “'de novo' UMP biosynthetic process”, “ribosomal large subunit assembly”, “structural constituent of ribosome”, “ribosomal large subunit biogenesis”, “RNA secondary structure unwinding”, “pseudouridine synthase activity”, “RNA processing, exonuclease activity”, “poly(A) RNA binding”
Organization of chromosomes	“Chromosome condensation”, “Condensed chromosome kinetochore”, “Spindle organization”
Cellular redox balance	"Oxidation-reduction process", "Glutathione transferase activity", "Glutathione metabolic process", “Methionine biosynthetic process”, "Cysteine biosynthetic process from serine"
Excretion processes	"Cytoplasmic vesicle", "Vacuolar transport", "Golgi apparatus", "Golgi stack", “Vacuole fusion, non-autophagic", "Endosome", "endopeptidase activity", "protein secretion", "protein transport", "proteasome complex"
