# Supplementary material for: Sexual reproduction during diatom bloom
Source: ISME Commun. 2025 Jan 7;5(1):ycae169. doi: 10.1093/ismeco/ycae169 (PMC11749564; doi:10.1093/ismeco/ycae169)
Supplement: List_of_supplementary_tables_and_figures_ycae169 [file list_of_supplementary_tables_and_figures_ycae169.pdf]

List of supplementary tables and figures:

Supplementary table 1: Metatranscriptomic samples metadata

Supplementary table 2: Gene annotations

Supplementary table 3: Read counts for the 25140 expressed *P. australis* genes

Supplementary table 4: Broad functions and related over-represented GO categories.

Supplementary table 5: Homology and expression levels (log2FC) *in vitro* (*P. multistriata*) and *in situ* (*P. australis*) of genes belonging to clusters 1, 3, 4 and 6. In vitro results from [17].

Supplementary table 6: functions over-represented in genes belonging to cluster 1 but down-regulated in vitro.

Supplementary table 7: functions over-represented in genes belonging to cluster 3 and 4 but up-regulated in vitro.

Supplementary figure 1: Map indicating the position of the sampling site in Western Europe.

Supplementary figure 2: Temporal evolution of the mean chlorophyll fluorescence per day between 2017-02-01 and 2017-05-30. The survey period is shown in black.

Supplementary figure 3: A. Relative abundances of the Arthropod's phylum community composition and B. Maxillopoda (copepods) phylum species community composition during

the 2017 monitoring (the category others regroup phylum and species lower than 2% of total abundance)
